# *Isospora basileuterusi* n. sp. (Apicomplexa: Eimeriidae) from the golden-crowned warbler *Basileuterus culicivorus* (Deppe) (Passeriformes: Parulidae) in South America

**DOI:** 10.1016/j.crpvbd.2022.100079

**Published:** 2022-01-31

**Authors:** Ericson R. Mello, Mariana S. Oliveira, Lucas de Assis S. Andrade, Sergian V. Cardozo, Águida A. Oliveira, Viviane M. Lima, Bruno P. Berto

**Affiliations:** a Programa de Pós-graduação em Ciências Veterinárias, Instituto de Veterinária, Universidade Federal Rural do Rio de Janeiro, Seropédica, Rio de Janeiro, Brazil; b Programa de Pós-graduação em Biologia Animal, Instituto de Ciências Biológicas e da Saúde, Universidade Federal Rural do Rio de Janeiro, Seropédica, Rio de Janeiro, Brazil; c Programa de Pós-Graduação em Biomedicina Translacional, Universidade do Grande Rio, Duque de Caxias, Rio de Janeiro, Brazil; d Departamento de Microbiologia e Imunologia Veterinária, Instituto de Veterinária, Universidade Federal Rural do Rio de Janeiro, Seropédica, Rio de Janeiro, Brazil; e Departamento de Biologia Animal, Instituto de Ciências Biológicas e da Saúde, Universidade Federal Rural do Rio de Janeiro, Seropédica, Rio de Janeiro, Brazil

**Keywords:** Coccidia, *Isospora*, Oöcyst morphology, Sequencing *cox*1, New species, Neotropical birds, Parulidae, Parque Nacional do Itatiaia

## Abstract

*Isospora basileuterusi* Mello & Berto n. sp. is described based on material from the golden-crowned warbler *Basileuterus culicivorus* (Deppe) captured in the Itatiaia National Park (Parque Nacional do Itatiaia), a conservation unit in south-eastern Brazil. Oöcysts of the new species are ellipsoidal to ovoidal, measuring on average 25.2 × 21.1 μm, with a smooth, bi-layered wall, *c.*1.6 μm thick. Micropyle and oöcyst residuum are both absent, but one to three polar granules are present. Sporocysts are ellipsoidal to lemon-shaped, measuring on average 15.3 × 9.5 μm, with a knob-like Stieda body and a trapezoidal sub-Stieda body. Sporocyst residuum is present, usually as a body of membrane-bound granules. Sporozoites are vermiform, with refractile bodies. Four of the 19 warblers captured (21%) were infected with the new species. Molecular analysis of the mitochondrial cytochrome *c* oxidase subunit 1 (*cox*1) gene revealed a similarity of 99.5% between the new species and *Isospora serinuse* Yang, Brice, Elliot & Ryan, 2015 from island canaries *Serinus canaria* (L.) in Western Australia. The oöcysts of *I. basileuterusi* n. sp. can be distinguished from the four other *Isospora* spp. recorded in hosts of the Parulidae, and from the molecularly most closely related species, by the typical ellipsoidal to lemon-shaped sporocysts, with small sub-Stieda body and a membrane-bound sporocyst residuum. Therefore, based on the morphological and molecular features, *I. basileuterusi* n. sp. is the fifth species described in a host of the family Parulidae and the first molecularly characterized *via* sequencing the *cox*1 gene.

## Highlights

• *Isospora basileuterusi* n. sp. is described from the golden-crowned warbler *Basileuterus culicivorus* (Deppe).
• Molecular characterization *via* sequencing of the mitochondrial cytochrome *c* oxidase subunit 1 gene is provided.
• 21% of the warblers captured in the Itatiaia National Park were positive for *Isospora basileuterusi* n. sp.
• *Isospora basileuterusi* n. sp. is the fifth species recorded in Parulidae, but the first molecularly identified.

## Graphical abstract

**Figure:**
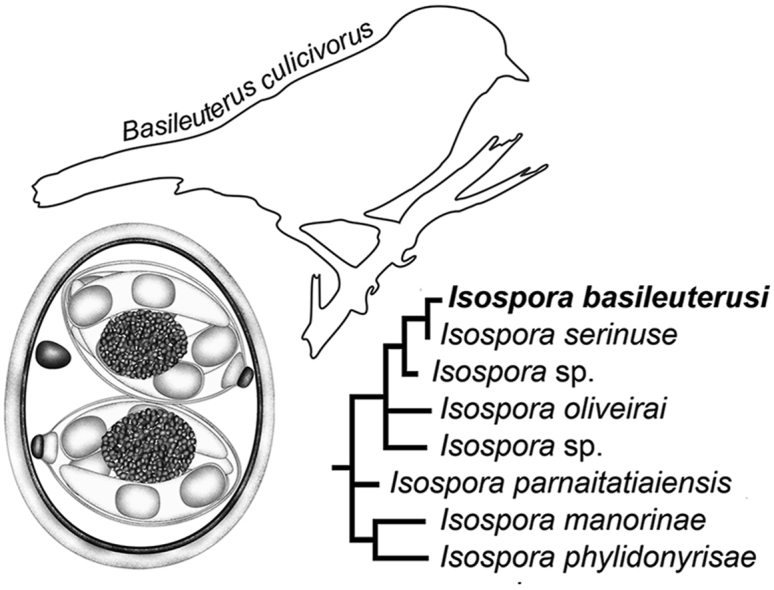

## 1 Introduction

Brazil is the second country in the Neotropical region with the highest number of bird species, with about 1971 species listed by the Brazilian Ornithological Records Committee (Pacheco et al., 2021), which corresponds to half of the of the diversity of the Neotropical avifauna (Dias, 1992). In relation to research on Neotropical birds, the study of their parasites has been highlighted for their association with ecology, biology and species conservation. Among their parasites, coccidian protozoans are important as a cause of morbidity and mortality, especially in captive birds or impacted environments, thus acting as ecological biomarkers (Berto & Lopes, 2020).

The golden-crowned warbler *Basileuterus culicivorus* (Deppe) is a passerine bird of the family Parulidae with a wide distribution in the Neotropical region (Sick, 1997; Pacheco et al., 2021). It has insectivorous eating habits and occupies the middle stratum of the ombrophilous forests (Marini & Cavalcanti, 1993; Lima & Manhães, 2009). The present study provides a description and molecular characterization of a new species of *Isospora* from golden-crowned warblers *B. culicivorus* captured in the Itatiaia National Park (Parque Nacional do Itatiaia), a conservation unit in south-eastern Brazil.

## 2 Materials and methods

### 2.1 Sample collection

A total of 9 expeditions were conducted between 2014 and 2019 in the Itatiaia National Park, a protected area with a high degree of vulnerability, located in the Serra da Mantiqueira on the border of the States of Rio de Janeiro, Minas Gerais and São Paulo (ICMBIO, 2021), in August (22°26′19″S, 44°37′23″W) and November (22°26′57″S, 44°36′25″W) 2014; March (22°27′38″S, 44°35′34″W) 2015; March (22°19′46″S, 44°32′11″W) and October (22°27′38″S, 44°35′34″W) 2016; July (22°26′15″S, 44°18′33″W) and November (22°26′57″S, 44°36′25″W) 2017; August (22°26′57″S, 44°36′25″W) 2018; March (22°26′17″S; 44°37′33″W) 2019. A total of 19 *B. culicivorus* were captured with mist nets. The birds were kept in individual boxes and faeces collected immediately after defecation. After identification to the species level, the bird was photographed and released, and stool samples were placed in centrifuge tubes containing 2.5% potassium dichromate (K_2_Cr_2_O_7_) solution at 1:6 (v/v).

### 2.2 Morphological analyses

Samples were examined at the Laboratório de Biologia de Coccídios, Universidade Federal Rural do Rio de Janeiro (UFRRJ). All samples were incubated at room temperature (25 °C) for 10 days or until *c.*70% of the oöcysts were sporulated. Oöcysts were isolated by flotation in Sheather’s sugar saturated solution (specific gravity: 1.20) and examined microscopically using the technique described by Duszynski & Wilber (1997) and Berto et al. (2014a). Morphological observations, line drawings, photomicrographs and measurements were made using an Olympus BX binocular microscope (Olympus Optical, Tokyo, Japan) equipped with a digital camera Eurekam 5.0 (BEL Photonics, Monza, Italy). Line drawings were edited using two software applications (Corel DRAW and Corel PHOTO-PAINT) from CorelDRAW® (Corel Draw Graphics Suite, Version, 2020; Corel Corporation, Canada). All measurements are in micrometres and are given as the range followed by the mean in parentheses.

### 2.3 Molecular data generation

An individual oöcyst was isolated from serial dilutions of the oöcysts in drops on a microscope slide using a sterile micropipette. This isolated oöcyst was resuspended in PBS and washed by centrifuging until the supernatant became clear (Dolnik et al., 2009). DNA was extracted from the oöcyst using the Qiagen DNeasy Blood and Tissue Kit (Qiagen, São Paulo, Brazil) according to the manufacturerʼs instructions. Four freeze-thaw cycles were applied prior to DNA extraction in order to achieve complete lysis of the oöcysts. PCR amplification of a partial fragment of the mitochondrial cytochrome *c* oxidase subunit 1 (*cox*1) gene (*c.*250 bp) was carried out using nested PCR, as previously described by Dolnik et al. (2009) and Yang et al. (2015). The external primers COIbF1 (5′-GWT CAT TAG TAT GGG CAC ATC A-3′) and COIbR1 (5′-CCA AGA GAT AAT ACR AAR TGG AA-3′), produced an amplicon of *c.*302 bp in size and the internal primes COIbF2 (5′-GGG CAC ATC ATA TGA C-3′) and COIbR2 (5′-ATA GTA TGT ATC ATG TAR WGC AA-3′) produced an amplicon of 257 bp in size. The PCR reaction contained 12.5 μl of GoTaq® G2 Hot Start Colorless Master Mix (Promega Labs, São Paulo, Brazil) (1 × ), 0.25 μl of each primer (0.2 μM), 9 μl of nuclease-free water and 3 μl of DNA (for the primary reaction) or 3 μl primary PCR product (for the secondary reaction). Both primary and secondary PCR amplifications were conducted using the same cycling conditions: 1 cycle at 94 °C for 5 min, followed by 35 cycles (94 °C for 30 s, 47 °C for 45 s, and 72 °C for 1 min) and a final extension step at 72 °C for 5 min. The amplicons from the second round PCRs were purified using the Qiagen MinElute PCR Purification (Qiagen, São Paulo, Brazil).

### 2.4 DNA sequence analyses

All PCR amplicons were sequenced using the PCR forward and reverse primers by Ludwig Biotechnology, where an ABI-Prism 3500 Genetic Analyzer (Applied Biosystems, Foster City, California) was used for Sanger sequencing. The results of the sequencing reactions were analyzed and edited using the program Chromas 2.6. The newly generated sequence was compared to those for *Isospora* spp. and other coccidian parasites available in the GenBank database using the Basic Local Alignment Search Tool (BLAST). The phylogenetic tree was constructed using the newly generated *cox*1 sequence aligned with sequences for 18 species of *Isospora* available on GenBank. Distance analyses and phylogenies were conducted using MEGA X (Kumar et al., 2018). Briefly, Sanger sequencing chromatogram files were imported into MEGA X and the nucleotide sequences were curated, analyzed, and aligned with reference sequences from GenBank using Clustal W (http://www.clustalw.genome.jp). Maximum likelihood (ML) and Neighbor-Joining (NJ) trees were constructed, and the distances computed using the Tamura-Nei method based on model selection using ModelTest in MEGA X. Bootstrap analyses were conducted using 1000 replicates to assess the reliability of inferred tree topologies.

## 3 Results

Nineteen *B. culicivorus* were examined and four (21%) were positive for coccidian oöcysts of a morphotype not reported in the scientific literature. These positive warblers were captured in November 2014 and August 2018 on a trail named “Trilha das Borboletas” (Trail of the Butterflies) (22°26′57″S, 44°36′25″W), and in March 2019 at the starting point of the “Travessia Ruy Braga” (Ruy Braga Crossing) (22°26′17″S; 44°37′33″W) in the Itatiaia National Park. This material is described below.

### 3.1 *Isospora basileuterusi* Mello & Berto n. sp.

#### 3.1.1 Taxonomic summary

*Type-host:Basileuterus culicivorus* (Deppe) (Passeriformes: Parulidae), warbler.

*Type-locality:* Parque Nacional do Itatiaia (22°26′57″S, 44°36′25″W), Brazil.

*Type-material:* Photosyntypes, line drawing and oöcysts in 2.5% K_2_Cr_2_O_7_ solution (Williams et al., 2010) are deposited and available (http://r1.ufrrj.br/labicoc/colecao.html) in the Parasitology Collection of the Laboratório de Biologia de Coccídios, at UFRRJ, under the repository number P-124/2021. Photographs of the type-host specimen (symbiotype) are deposited in the same collection.

*Site in host:* Unknown.

*Prevalence:* 21% (4 out of 19 birds examined).

*Representative DNA sequence:* One representative *cox*1 sequence was deposited in the GenBank database under the accession number OM025014.

*ZooBank registration:* To comply with the regulations set out in Article 8.5 of the amended 2012 version of the International Code of Zoological Nomenclature (ICZN, 2012) details of the new species have been submitted to ZooBank. The Life Science Identifier (LSID) of the article is urn:lsid:zoobank.org:pub:DC2F625C–B798-46DE-84F6–F6883A90E4A5. The LSID for the new name *Isospora basileuterusi* Mello & Berto n. sp. is urn:lsid:zoobank.org:act:26689BA2-AAC9-4E41-B90E-E40C5934137B.

*Etymology:* The specific epithet is derived from the genus name of the type-host.

#### 3.1.2 Description

**Fig. 1:**
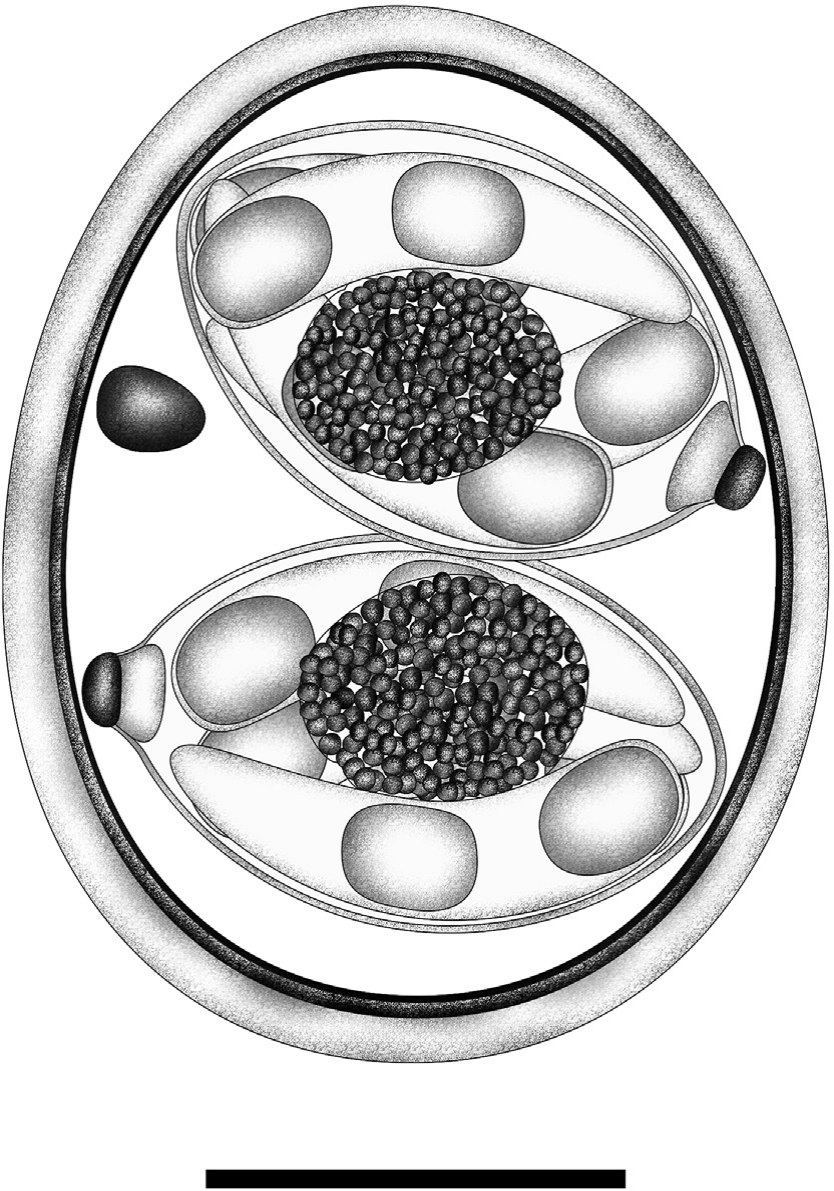
Composite line drawing of the sporulated oöcyst of *Isospora basileuterusi* from the golden-crowned warbler *Basileuterus culicivorus*. *Scale-bar*: 10 μm.

**Fig. 2:**
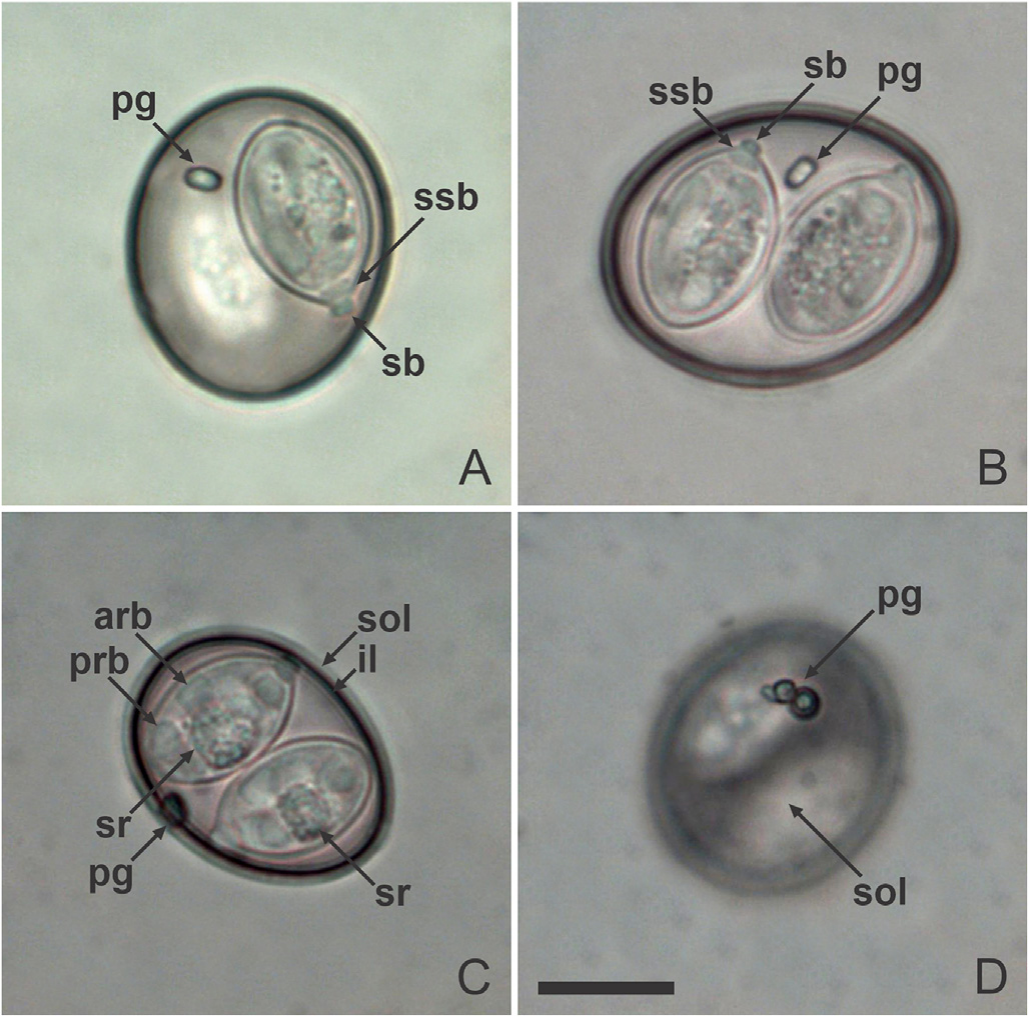
Photomicrographs of sporulated oöcysts of *Isospora basileuterusi* from the golden-crowned warbler *Basileuterus culicivorus*. *Abbreviations*: inner (il) and smooth outer (sol) layers of the oöcyst wall; polar granule (pg); Stieda (sb) and sub-Stieda bodies (ssb); sporocyst residuum (sr); posterior (prb) and anterior (arb) refractile bodies. All to same scale. *Scale-bar*: 10 μm.

[Based on 25 oöcysts and 25 sporocysts; Fig. 1, Fig. 2.] Oöcysts ellipsoidal to ovoidal, 22–28 × 17–23 (25.2 × 21.1); L/W ratio 1.1–1.3 (1.20). Wall bi-layered, 1.5–1.9 (1.6) thick, outer layer smooth. Micropyle and oöcyst residuum both absent, but one to three (usually one) polar granules present, 2.4–3.0 × 1.7–2.4 (2.7 × 2.0). Sporocysts 2, ellipsoidal to lemon-shaped, 14–17 × 8–11 (15.3 × 9.5); L/W ratio 1.4–1.8 (1.61). Stieda body present, knob-like, 0.9–1.1 × 1.7–2.1 (1.0 × 1.8); sub-Stieda present, trapezoidal, 1.1–1.7 × 2.5–2.9 (1.4 × 2.7); para-Stieda body absent; sporocyst residuum present, usually a distinctly ovoidal to ellipsoidal body consisting of numerous small granules that appear to be membrane-bound, 4.3–5.2 × 3.5–4.3 (3.9 × 4.8). Sporozoites 4, vermiform, with anterior and posterior refractile bodies and indiscernible nucleus.

#### 3.1.3 Remarks

To date, four *Isospora* spp. are recorded from warblers (Table 1, Table 2). The sizes of the oöcysts of all these species are reasonably compatible with *I. basileuterusi* n. sp.; however, these can be easily distinguished by a few characteristic features: the new species is the only one with ellipsoidal to lemon-shaped sporocysts, small sub-Stieda body and membrane-bound sporocyst residuum. Additionally, *I. basileuterusi* n. sp. does not have the typical characteristics of the other species, such as the absence of polar granules in *Isospora cardellinae* Salgado-Miranda, Medina, Zepeda-Velázquez, García-Conejo, Galindo-Sánchez, Janczur & Soriano-Vargas, 2016 and *Isospora celata* Berto, Medina, Salgado-Miranda, García-Conejo, Janczur, Lopes & Soriano-Vargas, 2014 (see Berto et al., 2014b; Salgado-Miranda et al., 2016), the presence of oöcyst residuum in *I. celata*, the compartmentalized sub-Stieda body in *Isospora orbisreinitas* Keeler, Yabsley, Adams & Hernandez, 2014 and the large and trapezoidal sub-Stieda body in *Isospora piacobrai* Berto, Flausino, Luz, Ferreira & Lopes, 2010 (see Berto et al. (2009); Keeler et al., 2014).

**Table 1:** Comparative morphological data for oöcysts of *Isospora* spp. recorded from warblers (Parulidae)

Species	Host	Shape	Size (μm)	Shape index	Polar granules	Oöcyst residuum	Wall (μm)	Micropyle	Reference
*Isospora basileuterusi* Mello & Berto n. sp.	*Basileuterus culicivorus* (Deppe)	Ellipsoidal to ovoidal	22–28 × 17–23 (25.2 × 21.1)	1.1–1.3 (1.20)	Present, 1–3 (usually one)	Absent	1.5–1.9 (1.6)	Absent	Present study
*Isospora cardellinae* Salgado-Miranda, Medina, Zepeda-Velázquez, García-Conejo, Galindo-Sánchez, Janczur & Soriano-Vargas, 2016	*Cardellina rubra* (Swainson)	Subspherical	23–28 × 23–27 (26.6 × 25.4)	1.0–1.1 (1.1)	Absent	Absent	1.2–1.4 (1.3)	Absent	Salgado-Miranda et al. (2016)
*Isospora celata* Berto, Medina, Salgado-Miranda, García-Conejo, Janczur, Lopes & Soriano-Vargas, 2014	*Leiothlypis celata* (Say)	Subspherical	27–30 × 25–28 (28 × 26)	1.0–1.1 (1.1)	Absent	Present	1.0–1.3 (1.2)	Absent	Berto et al. (2014)
*Isospora orbisreinitas* Keeler, Yabsley, Adams & Hernandez, 2014	*Basileuterus rufifrons* (Swainson)	Subspherical to ovoidal	21–28 × 19–25 (24.3 × 22.3)	1.0–1.3 (1.0)	Absent or present, 0–4	Absent	–	Absent	Keeler et al. (2014)
*Isospora piacobrai* Berto, Flausino, Luz, Ferreira & Lopes, 2010	*Geothlypis aequinoctialis* (Gmelin)	Subspherical to ovoidal	21–26 × 20–24 (23.5 × 21.6)	1.1–1.1 (1.1)	Present, 1	Absent	–	Absent	Berto et al. (2010)

**Table 2:** Comparative morphological data for sporocysts of *Isospora* spp. recorded from warblers (Parulidae)

Species	Host	Shape	Size (μm)	Shape index	Stieda body (μm)	Sub-Stieda body (μm)	Sporocyst residuum	Reference
*Isospora basileuterusi* Mello & Berto n. sp.	*Basileuterus culicivorus* (Deppe)	Ellipsoidal to lemon-shaped	14–17 × 8–11 (15.3 × 9.5)	1.4–1.8 (1.61)	Present, knob-like, 0.9–1.1 × 1.7–2.1 (1.0 × 1.8)	Present, trapezoidal, 1.1–1.7 × 2.5–2.9 (1.4 × 2.7)	Granules membrane-bound	Present study
*Isospora cardellinae* Salgado-Miranda, Medina, Zepeda-Velázquez, García-Conejo, Galindo-Sánchez, Janczur & Soriano-Vargas, 2016	*Cardellina rubra* (Swainson)	Ovoidal	18–20 × 11–13 (19.0 × 12.0)	1.6–1.8 (1.7)	Present, knob-like, (1.1 × 2.4)	Present, trapezoidal to rounded, sometimes with irregular base, (1.8 × 4.5)	Scattered spherules	Salgado-Miranda et al. (2016)
*Isospora celata* Berto, Medina, Salgado-Miranda, García-Conejo, Janczur, Lopes & Soriano-Vargas, 2014	*Leiothlypis celata* (Say)	Ovoidal	15–20 × 11–14 (18 × 13)	1.4–1.5 (1.4)	Present, knob-like, (1.0 × 2.5)	Present, irregular, barely discernible, (1.5 × 4.0)	Scattered spherules	Berto et al. (2014)
*Isospora orbisreinitas* Keeler, Yabsley, Adams & Hernandez, 2014	*Basileuterus rufifrons* (Swainson)	Ovoidal	12–19 × 10–14 (16.0 × 11.8)	1.0–1.9 (1.4)	Present, knob-like	Present, prominent, trapezoidal and compartmentalized	Many diffuse granules	Keeler et al. (2014)
*Isospora piacobrai* Berto, Flausino, Luz, Ferreira & Lopes, 2010	*Geothlypis aequinoctialis* (Gmelin)	Ovoidal	15–17 × 9–12 (15.8 × 10.5)	1.4–1.6 (1.5)	Present, knob-like and prominent, (1.0 × 1.7)	Present, large, trapezoidal and homogeneous, (2.3 × 4.8)	Granules of different sizes	Berto et al. (2010)

**Fig. 3:**
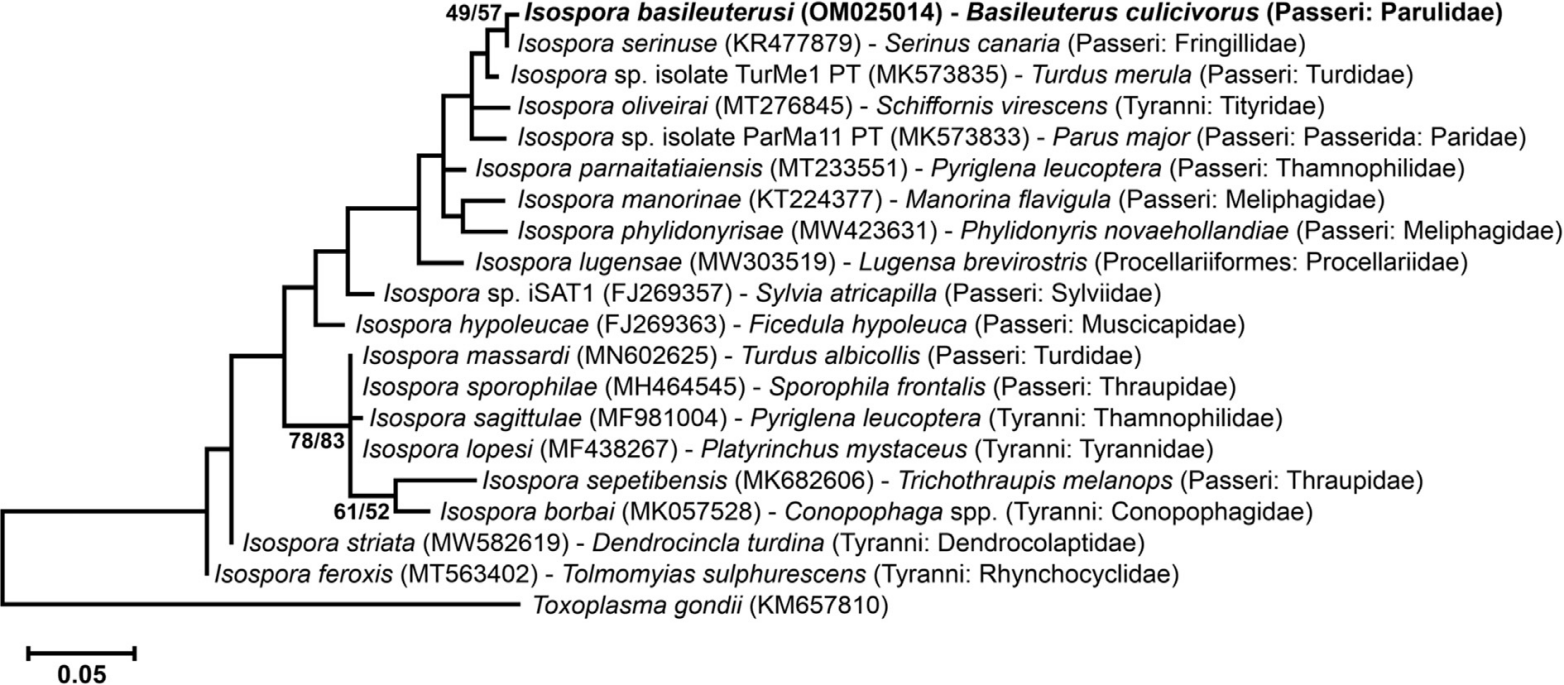
Maximum likelihood tree for *Isospora* spp. estimated from the *cox*1 sequences. Numbers at nodes represent bootstrap support (1000 replicates; only values > 50% shown) for Neighbor-Joining and Maximum Likelihood, respectively. The scale-bar represents the number of nucleotide substitutions per site.

*Isospora basileuterusi* n. sp. also differs morphologically from the molecularly most closely related *Isospora* spp. (Fig. 3), *Isospora serinuse* Yang, Brice, Elliot & Ryan, 2015 and *Isospora oliveirai* Ortúzar-Ferreira & Berto, 2020. The same characteristics typical of the new species, i.e. ellipsoidal to lemon-shaped sporocysts, small sub-Stieda body and membrane-bound sporocyst residuum, are not observed in *I. serinuse* and *I. oliveirai*.

### 3.2 Phylogenetic analysis

DNA amplification of the oöcyst of *I. basileuterusi* n. sp. showed a clear band of *c.*250 bp. Phylogenetic analysis included 18 sequences for avian *Isospora* spp. available on GenBank (Fig. 3). *Toxoplasma gondii* (Nicolle & Manceaux, 1908) was used as the outgroup. *Isospora basileuterusi* n. sp. was recovered in a clade with the highest similarity of 99.5% with *I. serinuse* from island canaries *Serinus canaria* (L.) in Western Australia (Yang et al., 2015). Furthermore, *I. basileuterusi* n. sp. was closely related (95–97%) to *I. oliveirai* from the greenish schiffornis *Schiffornis virescens* (Lafresnaye) in south-eastern Brazil and *Isospora* spp. recovered from thrushes (Turdidae) and tits (Paridae) in Czech Republic (Trefancová & Kvičerová, 2019; Ortúzar-Ferreira et al., 2020).

## 4 Discussion

Duszynski & Wilber (1997) compiled almost all taxonomic studies of coccidia of passerines and advised that new coccidian identifications should be based on comparative morphology between coccidian species recorded in the same host family. In this sense, the morphotype observed from the golden-crowned warblers in this study, *I. basileuterusi* n. sp., was compared with the four recorded coccidian species of Parulidae, as shown in Table 1, Table 2. *Isospora basileuterusi* n. sp. differs in several characteristic features, but can be mainly differentiated from the others by the typical lemon-shape of its sporocysts.

The host of the new coccidian species described here, the golden-crowned warbler *B. culicivorus*, has a wide distribution in the Neotropical region, from Mexico to southern South America (Pacheco et al., 2021). However, according to BirdLife International (2021) this species is the stripe-crowned warbler, which is restricted to Mexico and Central America, not occurring in Brazil. This misinformation is due to species/subspecies status within the genus *Basileuterus* Cabanis. Birdlife have reclassified some subspecies of *B. culicivorus* to the species level, such as *Basileuterus culicivorus auricapilla* (Swainson, 1838) which has been reclassified to the level of species, as *Basileuterus auricapilla* Swainson, 1838. Therefore, this study followed the name listed by the Brazilian Ornithological Records Committee (Pacheco et al., 2021); however, it is noteworthy that the bird specimen in this study is identified as *B. auricapilla* by BirdLife International (2021). Anyway, regardless of the specific identification within *Basileuterus*, due to the wide geographical distributions of warblers (Parulidae) in the Americas, their coccidian species must be equally distributed throughout the Neotropical region.

In the present study, the molecular identification of *I. basileuterusi* n. sp. was performed using the *cox*1 gene, which is considered to be the gene with the highest resolution in detecting recent speciation events (Barta, 2001; Ogedengbe et al., 2011). In fact, the 250 bp *cox*1 gene sequence was not 100% similar to any other deposited on GenBank, contrary to what occurs with ribosomal gene sequences that are more conserved and more suitable for phylogenetic studies of families and orders (Genovez-Oliveira et al., 2020). On the other hand, the region of the *cox*1 sequenced for *I. basileuterusi* n. sp. did not provide conclusive results related to ancestry, as linked to host family, biogeographical region, morphology/biology of the coccidian species, etc. (Fig. 3). This is also observed when *Isospora* spp. amplified and sequenced with the primers (Dolnik et al., 2009) used in the present study are exclusively included in the phylogeny, as in the studies of Yang et al. (2015) and Silva-Carvalho et al. (2018). Perhaps the short sequence of only 250 bp did not allow greater resolution in the phylogenetic study; in this case, sequences with more than 600 bp from other regions of the *cox*1 gene, such as those generated by the JAV primer (Genovez-Oliveira et al., 2020), would show better phylogenetic estimations in the future. Fatally, in the present study these JAV primers were not successful in amplifying the samples; however, it has been shown in any case that mitochondrial genes, such as *cox*1, are better suited to work with individual oöcysts, as the number of copies of mitochondrial DNA is far greater than the number of copies of nuclear DNA, thus favoring the amplification of mitochondrial genes (Dolnik et al., 2009).

## 5 Conclusion

The comparison of *I. basileuterusi* n. sp. with *Isospora* spp. described from Neotropical warblers clearly supports the designation as a unique species. Therefore, *I. basileuterusi* is considered as new to science, which is the fifth species described in a host of the family Parulidae and the first molecularly characterized *via* sequencing the *cox*1 gene.

## Funding

This study was supported by 10.13039/501100003593Conselho Nacional de Desenvolvimento Científico e Tecnológico, 10.13039/501100002322Coordenação de Aperfeiçoamento de Pessoal de Nível Superior and 10.13039/501100004586Fundação Carlos Chagas Filho de Amparo à Pesquisa do Estado do Rio de Janeiro. MSO has a post-doctoral scholarship from FAPERJ (Grant/Award Number: E−26/204.228/2021). LASA has a scholarship from CAPES (Grant/Award Number: 001). BPB has fellowships from CNPq (Grant/Award Number: 303899/2019-0) and from FAPERJ (Grant/Award Number: E−26/202.797/2019).

## Ethical approval

Field-collecting permits were issued by SISBIO/ICMBio (licenses 45,200; 49,605; 54,951; 70,132), CEUA/UFRRJ (protocols IV-036/2014; ICBS-008/2015; IV-6606250616) and CEUA/UNIGRANRIO (protocol 021/2019). All applicable institutional, national and international guidelines for the care and use of animals were followed.

## CRediT author statement

The study was designed by SVC, VML and BPB. Field work was performed by MSO, LASA and BPB. Laboratory procedures for maintenance, recovery, measurements, photomicrographs and isolation of oöcysts were performed by MSO and LASA. DNA extraction, amplification and sequencing were performed by ERM, AAO and VML. BPB analyzed the data and drew the coccidian oöcyst. The manuscript was written by ERM and BPB and subsequently revised by all other authors. All authors read and approved the final manuscript.

## Data availability

Photosyntypes, line drawing, and oöcysts in 70% ethanol are deposited and available (http://r1.ufrrj.br/labicoc/colecao.html) in the Parasitology Collection of the Laboratório de Biologia de Coccídios, at UFRRJ, under the repository number P-124/2021, along with the photographs of the type-host specimen (symbiotype). The generated sequence for *I. basileuterusi* n. sp. is deposited in the GenBank database under the accession number OM025014.

## Declaration of competing interests

The authors declare that they have no known competing financial interests or personal relationships that could have appeared to influence the work reported in this paper.
